# Supplementary figures and images for: Development and validation of a set of novel and robust 4-lncRNA-based nomogram predicting prostate cancer survival by bioinformatics analysis
Source: PLoS One. 2021 May 4;16(5):e0249951. doi: 10.1371/journal.pone.0249951 (PMC8096091; doi:10.1371/journal.pone.0249951)

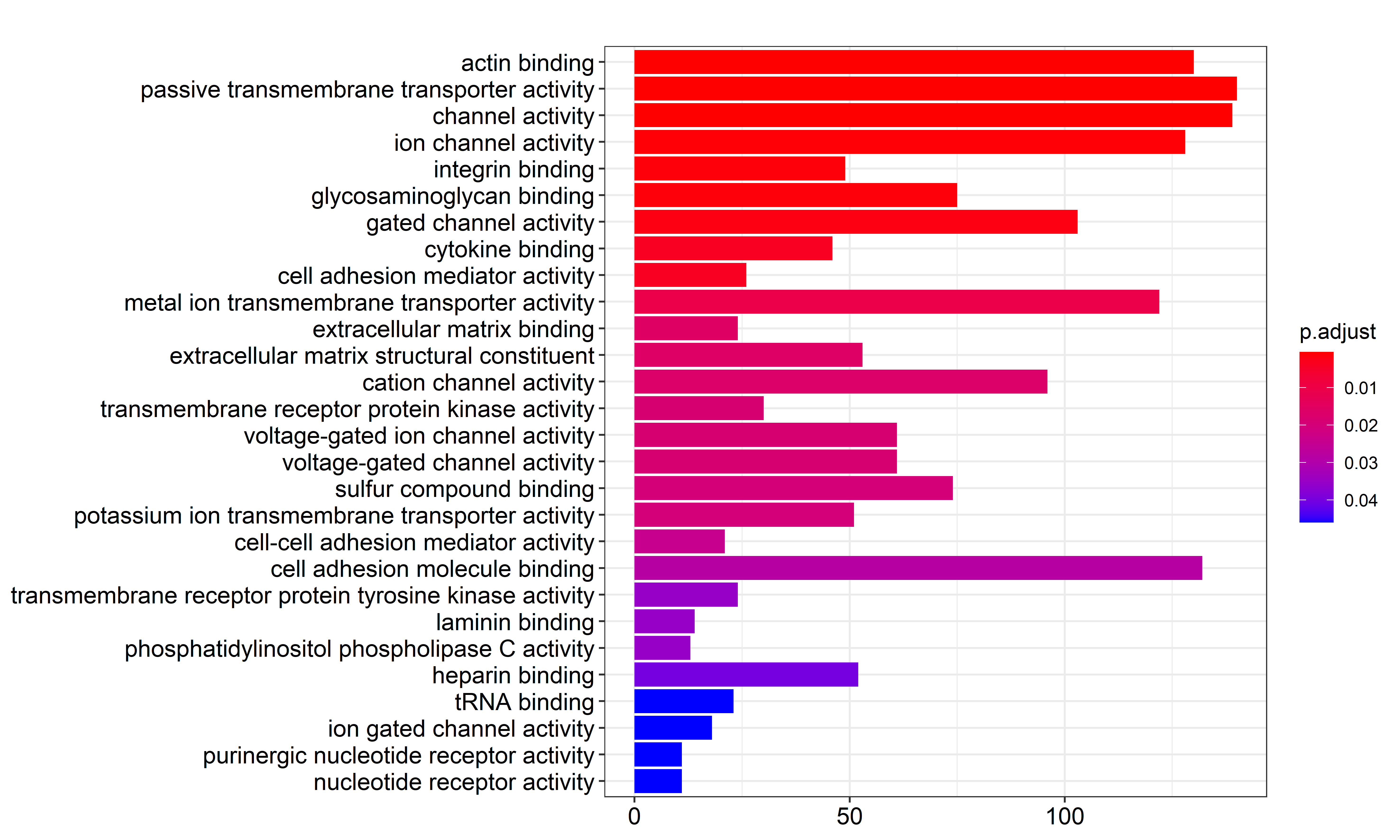

Supplement: S2 Fig — (TIFF) [file pone.0249951.s002.tiff]

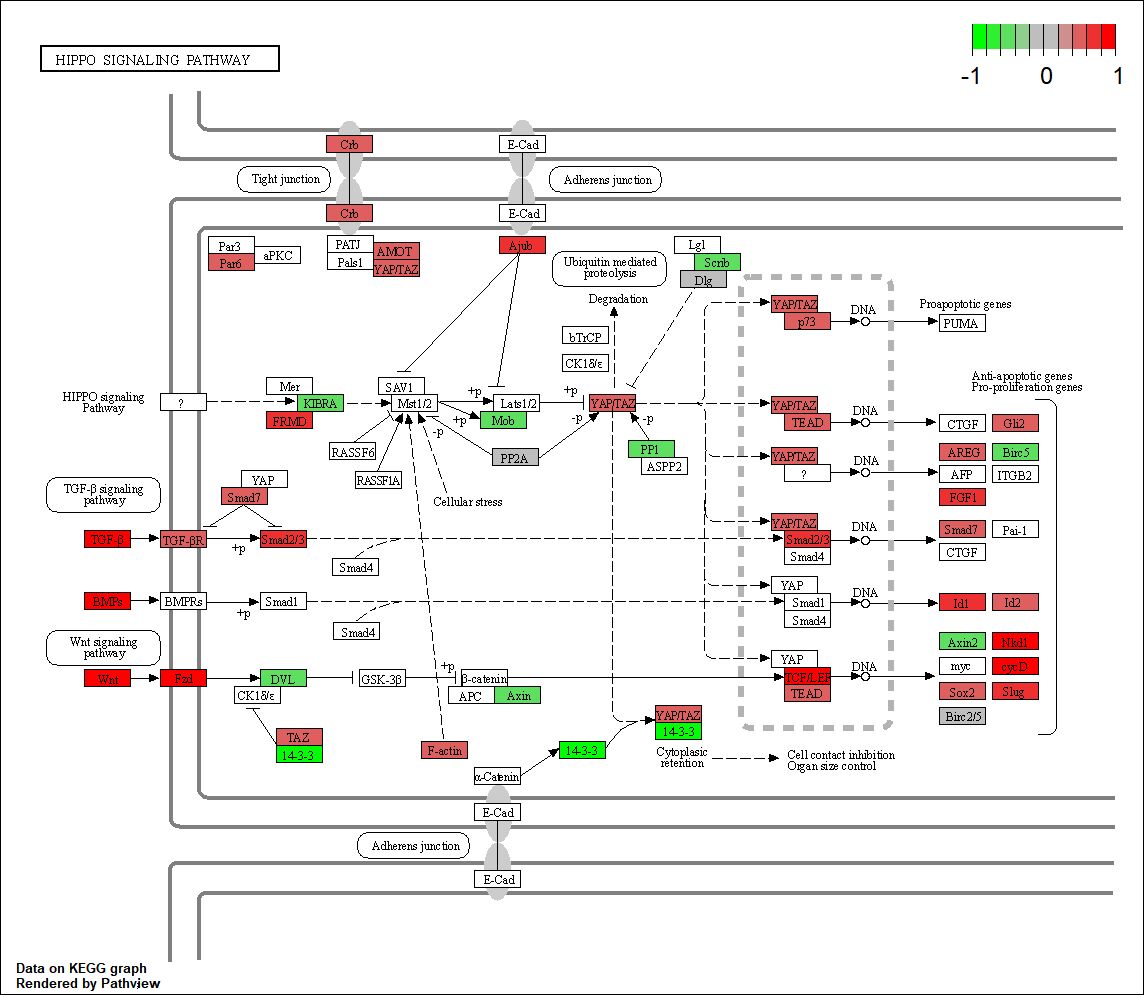

Supplement: S3 Fig — (A) Hippo signaling pathway, (B) Focal adhesion and (C) Cell adhesion molecules (CAMs). (ZIP) [file pone.0249951.s003.zip › S3A_Fig.jpg]

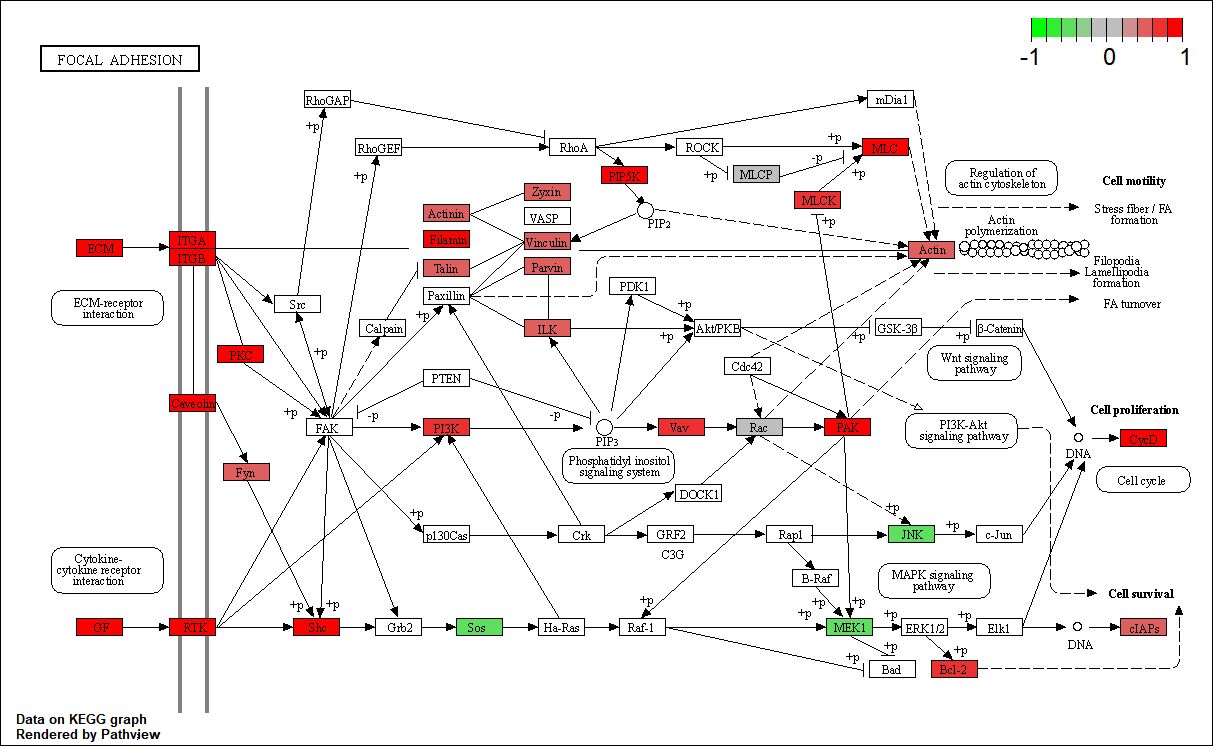

Supplement: S3 Fig — (A) Hippo signaling pathway, (B) Focal adhesion and (C) Cell adhesion molecules (CAMs). (ZIP) [file pone.0249951.s003.zip › S3B_Fig.jpg]

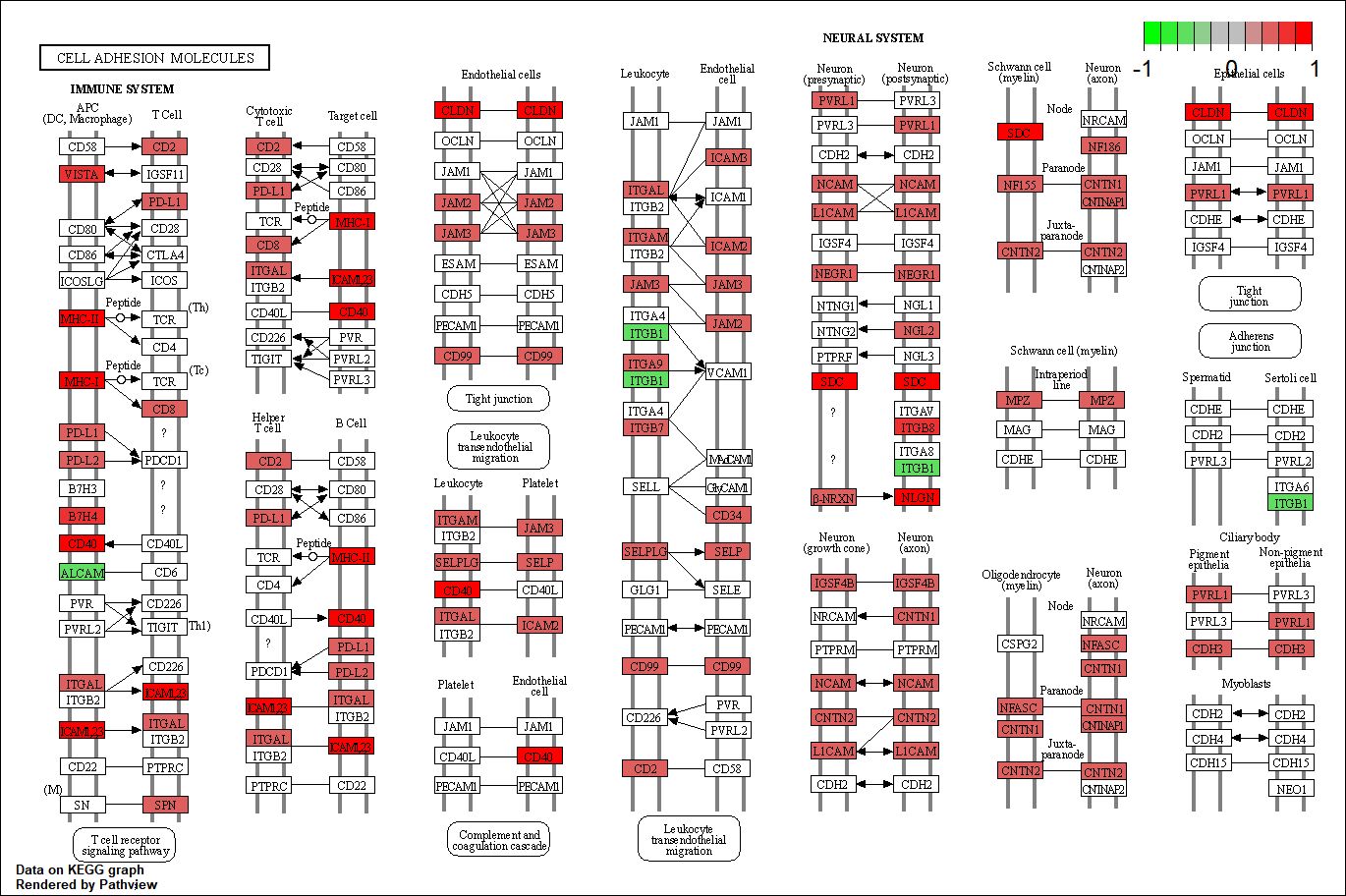

Supplement: S3 Fig — (A) Hippo signaling pathway, (B) Focal adhesion and (C) Cell adhesion molecules (CAMs). (ZIP) [file pone.0249951.s003.zip › S3C_Fig.jpg]

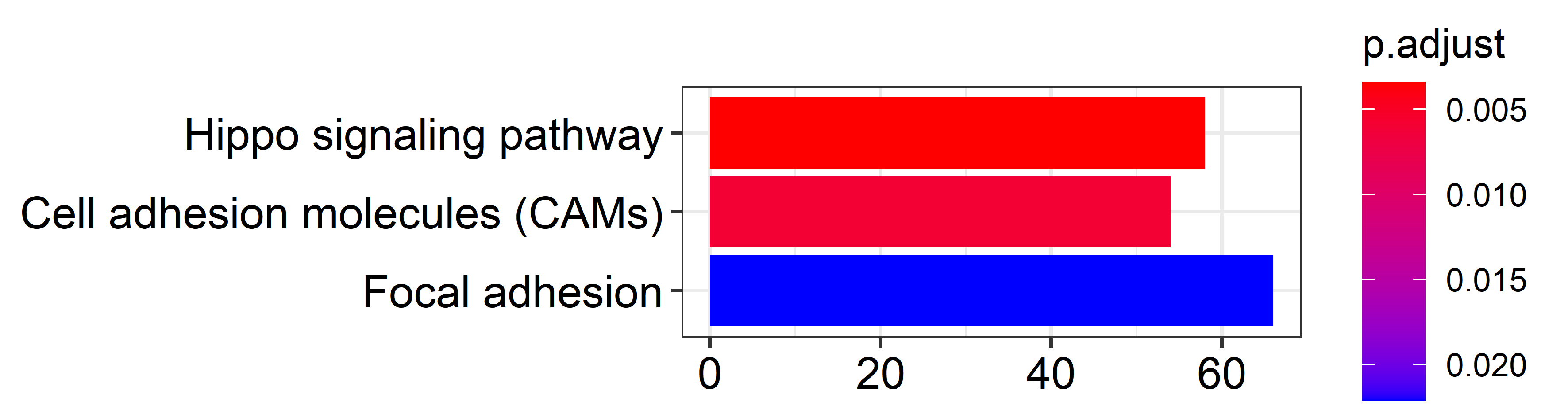

Supplement: S4 Fig — (TIFF) [file pone.0249951.s004.tiff]
