# Supplementary material for: Development and validation of a set of novel and robust 4-lncRNA-based nomogram predicting prostate cancer survival by bioinformatics analysis
Source: PLoS One. 2021 May 4;16(5):e0249951. doi: 10.1371/journal.pone.0249951 (PMC8096091; doi:10.1371/journal.pone.0249951)
Supplement: Attachment — Submitted filename: Response to Reviewers_Feb15.docx [file pone.0249951.s012.docx]

Dear Dr. Hiromu Suzuki

Thank you very much for your decision letter and advice on our manuscript (Manuscript PONE-D-20-27863) entitled “Development and validation of a set of novel and robust 4-lncRNA-based nomogram predicting prostate cancer survival by bioinformatics analysis”. We also thank the reviewers for the constructive comments and suggestions. We have revised the manuscript accordingly, and all amendments are indicated by red font in the revised manuscript. In addition, our point-by-point responses to the comments are listed below this letter.

We hope that our revised manuscript is now acceptable for publication in your journal and look forward to hearing from you soon.

With best wishes,

Yours sincerely,

Xuefeng Zhang

First of all, we would like to express our sincere gratitude to the reviewers for their constructive and positive comments.

**Replies to Reviewer 1**

Reviewer #1: The manuscript by Zhang, et al. uses the TCGA data to identify differentially expressed lncRNA between cancerous and non-cancerous tissues. In their analysis, they identified four lncRNA’s (2 protective and 2 harmful) that help improve stratification of overall survival from prostate cancer. Evaluating their data, it appears that there were only 9 deaths amongst the 397 patients who were included in the study. Therefore, since they are only evaluating overall survival, and not biochemical recurrence, distant metastasis, or progression of disease, how their results can be meaningful in making a clear utility of the four lncRNA’s.

The limitations of their study are not discussed at all. They also make overstatements that their findings can be used to determine need for adjuvant therapy. However, without validation and evaluation of other parameters, besides overall survival, they cannot make such lofty claims.

**Response:** We added gene sequencing and clinical data from ICGC database to conduct additional analysis and verification of the prediction performance of our prediction model. Because the mortality rate for prostate cancer in the United States is so low, only nine of the 397 Americans from the TCGA database previously included in the study died. Because this study evaluated overall survival, we added 378 patients from the ICGC database from China, 34 of whom died. Thus, the reliability of this study is further increased. We have added a discussion of the limitations of this study.

**Replies to Reviewer 2**

Reviewer #2: 1、The as-constructed lncRNAs-based signature in this study can serve as a novel tool and shed more lights on PCa diagnosis and treatment. The 4 lncRNAs–based signature is constructed to predict the OS for PCa through bioinformatic analysis. This prediction signature outstandingly improves the Gleason score performance in distinguishing PCa, and it can also be used to discriminate PCa (Gleason > 7).
2、It is better to add other databases analysis and related function experiments.
3、A few sentence typos，such as line78 4-lncrNA etc.

**Response:**

1. We added gene sequencing and clinical data from ICGC database to conduct additional analysis and verification of the prediction performance of our prediction model.

2. In view of the expression errors in our paper you proposed, we have also made corresponding modifications.

3. Correction has been made in the revised manuscript
